# Distinct effects of obesity and diabetes on the action potential waveform and inward currents in rat ventricular myocytes

**DOI:** 10.1042/CS20242144

**Published:** 2025-01-15

**Authors:** Anatoliy Shmygol, Gilles Bru-Mercier, Ahmed S. Sultan, Frank C. Howarth

**Affiliations:** 1 Department of Physiology, College of Medicine and Health Sciences, United Arab Emirates University, P.O. Box 15551, Al Ain, Abu Dhabi, UAE; 2 Zayed Center for Health Sciences, United Arab Emirates University, Al Ain, Abu Dhabi, UAE

**Keywords:** action potential, type 2 diabetes, voltage-gated Ca^2+^current, voltage-gated Na^+^current, Zucker rat

## Abstract

Obesity is a significant global health challenge, increasing the risk of developing type 2 diabetes mellitus (T2DM) and cardiovascular disease. Research indicates that obese individuals, regardless of their diabetic status, have an increased risk of cardiovascular complications. Studies suggest that these patients experience impaired electrical conduction in the heart, although the underlying cause—whether due to obesity-induced fat toxicity or diabetes-related factors—remains uncertain. This study investigated ventricular action potential parameters, as well as sodium (I_Na_) and calcium (I_Ca_, _L_) currents, in Zucker fatty (ZF) rats and Zucker diabetic fatty (ZDF) rats, which serve as models for obesity and T2DM, respectively. Ventricular myocytes were isolated from 25- to 30-week-old Zucker rats. Resting and action potentials were recorded using a β-escin perforated patch clamp, while I_Na_ and I_Ca,L_ were assessed with whole-cell patch clamp methods. ZF rats exhibited higher excitability and faster upstroke velocity with greater I_Na_ density, whereas ZDF rats showed decreased I_Na_ and slower action potential upstroke. No differences in I_Ca,L_ density or voltage sensitivity were found among the groups. In summary, obesity, with or without accompanying T2DM, distinctly impacts the action potential waveform, I_Na_ density, and excitability of ventricular myocytes in this rat model of T2DM.

## Introduction

Diabetes mellitus (DM) is the seventh leading cause of death worldwide. In 2017, an estimated 451 million adults aged 18–99 years had DM, which is expected to increase to nearly 700 million by 2045 [1–3]. Obesity, on the other hand, has almost tripled since 1975. Obese people are more likely to develop metabolic syndrome, insulin resistance and type 2 diabetes mellitus (T2DM) [2,4]. The term ‘diabesity’ was introduced to highlight the strong link between obesity and diabetes as the majority of individuals with diabetes are obese or overweight [5]. However, although most individuals with T2DM are obese, not all of them develop T2DM. Studies in non-diabetic obese patients suggest that obesity, independent of diabetes-related complications, significantly increases the risk of developing cardiovascular disease [6].

Cardiovascular disease is a leading cause of morbidity in obese individuals with T2DM. Previous studies have reported a multitude of vascular abnormalities, aberrant heart rhythm and defective electro-mechanical coupling in patients with diabetes [3,5,6].

Ethical constraints and practical limitations in studying T2DM in humans make it necessary to use animal models to explore the disease’s pathogenesis and the pathophysiological mechanisms underlying its complications. Although animal models cannot replicate all aspects of human disease, the Zucker diabetic fatty (ZDF) rat has garnered significant interest as one of the closest approximations to the human condition [7]. The original Zucker fatty (ZF) rat was found to harbour a spontaneous missense mutation designated ‘fatty’ (fa) in the leptin receptor gene. This results in the loss of function of this gene. As a result, homozygous animals with the (fa/fa) mutation develop hyperphagia, severe hyperlipidaemia and obesity without hyperglycaemia. Heterozygous (fa/+) animals do not show hyperphagia and remain lean (ZL rats) [8,9]. Subsequent observations revealed that, on rare occasions, the ZF male rats developed severe hyperglycaemia and high insulin resistance. Inbreeding these animals for ten generations resulted in the development of a strain of fatty rats consistently demonstrating a severe hyperglycaemic phenotype [10,11]. The resulting three strains of Zucker rats, namely, ZL, ZF and ZDF, provide a convenient model for investigating the cardiovascular effects of obesity separately from those caused by a combination of obesity and T2DM [12–14]. Initial research has suggested that ZDF rats do not develop cardiovascular disease spontaneously [15]. Subsequent studies, however, have uncovered a variety of cardiovascular abnormalities in this animal model of T2DM [12–14,16].

Similar to humans, diabetic animals often exhibit electrical remodeling of the myocardium, characterized by prolonged ventricular action potentials, impaired contractility, and abnormal conduction velocity [12,16,17]. Extensive electrophysiological studies in human and animal myocardium have attributed diabetes-induced prolongation of the ventricular action potentials to a decrease in outward K^+^ currents and possible upregulation of inward Ca^2+^ current [17–19]. A well-documented downregulation of voltage-gated potassium currents, particularly the transient outward current, is considered a major mechanism underlying the prolongation of ventricular action potentials in rats with chronic diabetes mellitus [20–24]. The involvement of voltage-gated Ca²^+^ and Na^+^ currents in the prolongation of cardiac action potentials remains unclear, with different studies presenting contradictory findings [17,19,25,26].

Decreased conduction velocity in diabetic myocardium is thought to be, at least in part, due to malfunctioning gap junctions between neighbouring myocytes. This dysfunction is believed to result from an imbalance between phosphorylated and dephosphorylated connexin 43 (Cx43) and/or the redistribution of Cx43 [27,28]. Olsen et al. have described impaired conduction velocity in the myocardium of ZDF rats, accompanied by increased lateralization of Cx43 [29]. The authors concluded that the observed lateralization of Cx43 was not the sole cause of impaired conduction, suggesting that additional mechanisms were also involved. An important determinant of cardiac impulse propagation is the large and rapidly activating sodium current [30,31]. Aberrant inward currents with a concomitant slow propagation velocity of ventricular action potential increase the risk of developing re-entry arrhythmias, while the appearance of steady-state current through sodium channels may generate early after depolarizations leading to *torsades des pointes* and eventually to ventricular fibrillation [31–35]. In this study, we tested the hypothesis that obesity and diabetes modulate the ventricular action potential differentially by selectively enhancing or reducing the fast sodium current. We found that, in diabetic animals, the rate of rise of the ventricular action potential was significantly reduced compared with that in fatty non-diabetic animals. This reduction was accompanied by a marked decrease in I_Na_, suggesting a causative relationship.

## Materials and methods

### Animals

Experiments were performed on 16 ZDF, 16 ZF and 13 ZL male rats (Charles River Laboratories, Margate, Kent, UK), with the experiments beginning at 195 days of age. Rats were maintained under a 12-hour light/12-hour dark cycle with free access to water and a standard chow diet. Body weight, heart weight and non-fasting blood glucose (OneTouch Ultra 2, LifeScan) were measured immediately before each cell isolation. Insulin concentration in blood plasma was measured using a commercially available rat insulin ELISA kit (10-1250-01, Mercodia Rat Insulin ELISA).

### Isolation of ventricular myocytes

Left ventricular myocytes were isolated from the rats according to previously described techniques [35,36]. In brief, the animals were euthanized using a guillotine, and hearts were removed rapidly and mounted for retrograde perfusion according to the Langendorff method. Hearts were perfused at a constant flow of 8 ml/g heart/min and at 36–37°C with cell isolation solution containing in mmol/l: 130 NaCl, 5.4 KCl, 1.4 MgCl_2_, 0.75 CaCl_2_, 0.4 NaH_2_PO_4_, 5.0 HEPES, 10 glucose, 20 taurine and 10 creatine (pH 7.3). Perfusion flow rate was adjusted to allow for differences in heart weight between animals. When the heart had stabilized, perfusion was continued for 4 min with Ca^2+^-free cell isolation solution containing 0.1 mmol/l EGTA and then for 6 min with cell isolation solution containing 0.05 mmol/l Ca^2+^, 0.75 mg/ml collagenase type 1 (Worthington Biochemical Corp, Lakewood, NJ, U.S.A.), and 0.075 mg/ml type XIV protease (Sigma, Taufkirchen, Germany). Left ventricle tissue was excised from the heart, minced, and gently shaken for 4 min at 36-37 °C in collagenase-containing isolation solution supplemented with 1 mg/ml fatty acid-free bovine serum albumin. The cell suspension was then filtered using a 300-micron nylon mesh (Cadisch Precision Meshes, U.K.). The filtrate was centrifuged at 1000 rpm for 60 sec. The supernatant was then removed, and the cell pellet was resuspended in a cell isolation solution containing 0.75 mmol/l Ca^2+^. The last step was repeated four times. Cells were used within 6–8 hours after the isolation. During this time, the cells retained their brick-like shape with sharp edges and a clear sarcomere patterning.

### Action potential measurement

A drop of cell suspension was added to the bath chamber mounted on the stage of an inverted microscope (IX-71, Olympus, U.K.) and left undisturbed for 5–10 min to allow the myocytes to adhere to the bottom of the chamber. Cells were then superfused at 2 ml/min flow rate with Tyrode’s solution containing (in mM) NaCl 140, KCl 5, CaCl_2_ 1.8, MgCl_2_ 1.0, NaH_2_PO_4_ 0.33, HEPES 10.0 and glucose 10.0, with pH adjusted to 7.4 using 1M NaOH. The perforated-patch current-clamp technique was used for recording action potentials with 50 µM β-escin in the pipette solution as a perforating agent [37]. The patch pipettes were pulled from 1.2 mm borosilicate capillaries (Sutter Instruments, U.S.A.) using a two-stage puller (Model PP-830, Narishige, Japan) and fire-polished to facilitate the giga-seal formation. The polished pipettes had a 2–2.5 MΩ resistance when filled with the internal pipette solution. The pipette solution for these experiments was composed of the following (in mM): KCl 130, CaCl_2_ 0.5, MgCl_2_ 1.0, Mg-ATP 0.5, EGTA 5.0 and HEPES 10.0. The pH was adjusted to 7.3 with 1 M KOH. An appropriate quantity of the 50 mM β-escin stock solution was added to the pipette solution just before the experiment to achieve a 50 µM final concentration. The presence of β-escin in the pipette did not interfere with the seal formation. After obtaining a high-resistance (2–5 GΩ) seal, the membrane penetration usually started within 40–60 seconds, and a stable access resistance of 25–40 MΩ was obtained within 10–15 min. Cells that did not achieve the required level of access resistance were excluded from further experimentation.

An Axopatch 200B amplifier (Molecular Devices, Sunnyvale, CA, U.S.A.) was used for perforated-patch current-clamp recording of cardiac action potentials. The resting potential was shifted to –80 mV by applying a negative holding current after recording the existing resting potential (RP) and determining the stimulation threshold by applying 1 ms current stimuli of progressively increasing amplitude until an action potential was triggered. The stimulation current amplitude was increased 1.5 times above the threshold and used to elicit action potentials in all subsequent recordings. The stimuli were generated, and the resulting action potentials were recorded using a Digitata 1440A digitizer controlled by Clampex 10.6 data acquisition software (Molecular Devices, Sunnyvale, CA, U.S.A.). Four series of 100 stimuli were applied to each cell at frequencies of 1, 2, 5 and 10 Hz. Several parameters of the action potentials were extracted for further analysis using Clampfit 10.6. The parameters used for comparison of action potentials between the three groups of animals are presented in Table 1. The upstroke velocity (dV/dt_max_) of the action potential at each stimulation frequency was calculated in Clampfit as the maximum slope of the rising phase of the action potential. All experiments were performed at room temperature.

**Table 1:** Electrophysiological parameters of ventricular myocytes from the ZL, ZF and ZDF rats.

Action potential parameters (mean ± SEM)	ZL (*n* = 15; *N* = 6) ^a^	ZF (*n* = 15; *N* = 8)	ZDF (*n* = 13; *N* = 6)
Resting potential (RP) ^b^ Holding current at –80 mV	–63.6 ± 1.0 mV –0.134 ± 0.048 nA	–68.5 ± 1.3 mV –0.169 ± 0.022 nA	–63.5 ± 1.2 mV –0.156 ± 0.051 nA
Threshold potential Action potential amplitude	–50.5 ± 1.0 mV 128.2 ± 3.0 mV	–53.8 ± 1.5 mV 128 ± 2.0 mV	50.3 ± 1.0 mV 128 ± 2.5 mV
APD at 50% repolarization APD at 90% repolarization	9.2 ± 1.7 ms 31.7 ± 5.1 ms	15.5.3 ± 3.0 ms 49.0 ± 6.2 ms	12.2.9 ± 3.4 ms 45.0 ± 10.1 ms

^a^The *n* numbers refer to the number of cells; *N* number refers to the number of rats.

^b^Measured in each cell before applying the holding current required to set the RP at –80 mV.

### Sodium current recording

The experiments were performed simultaneously with the above current-clamp experiments on a second electrophysiology rig assembled around a Nikon Diaphot 300 microscope (Nikon, U.S.A). Voltage-gated Na^+^ current was recorded from isolated myocytes in whole-cell configuration of the patch clamp technique as previously described [36,38]. A 4 mM Na^+^ concentration was used in both the bath and the pipette for these experiments. The reduced Na^+^ concentration helped to avoid ‘voltage escape’ and achieved an adequate voltage clamp of large and fast current [36]. Cells were initially superfused with normal Tyrode’s solution as described above. After seal formation, the bath perfusion was switched to I_Na_ solution of the following composition (in mM): NaCl 4, TEACl 110, CsCl 4, CaCl_2_ 0.1, MgCl_2_ 1.0, HEPES 10.0 and glucose 10.0, with pH adjusted to 7.4 using 1M CsOH. To inhibit currents through the L-type Ca^2+^ channels, 15 µM verapamil was added to this solution. The pipettes were pulled from filamented BF150-86-10 borosilicate glass on a processor-controlled Flaming–Brown micropipette puller (P-97, Sutter Instrument, U.S.A.). The pipette resistance was 1.6–2.0 MΩ when filled with an internal solution containing (in mM) CsCl (120), NaCl (4.0), MgCl_2_ (1.0), Mg-ATP (2), EGTA (5.0) and HEPES (10.0). The pH was adjusted to 7.3 with 1 M CsOH. The pipettes were positioned and sealed to myocytes using a motorised micromanipulator (PatchStar, Scientifica, U.K.). A seal resistance exceeding 1 GΩ was obtained by applying a gentle negative pressure. The membrane under the pipette was ruptured by strong suction to gain access to the cell interior. After achieving a whole-cell configuration, the cell was dialyzed with the above solution for at least 5 min before initiating the voltage clamp recording. Access resistance was between 4 and 7 MΩ. Up to 75% of this was electronically compensated to improve the speed and reduce the voltage clamp error. An Axopatch 200B amplifier was used for whole-cell patch clamp recording. The voltage clamp protocols were generated, and currents were recorded using a Digidata 1550 digitizer (Molecular Devices, U.S.A.) and Clampex 10.6 software. The current output was filtered at 5 kHz using a four-poll Bessel filter on the Axopatch 200B and sampled at 20 kHz. The cells were held at –80 mV throughout the experiment. The ‘I_Na_–voltage’ relationship was measured using 100 ms pulses ranging from –80 to +15 mV after a 100 ms pre-pulse to –120 mV. Cells showing a leak current over 150 pA or signs of ‘voltage escape’ were excluded from further analysis.

### L-type Ca^2+^ current recording

**Figure 1:**
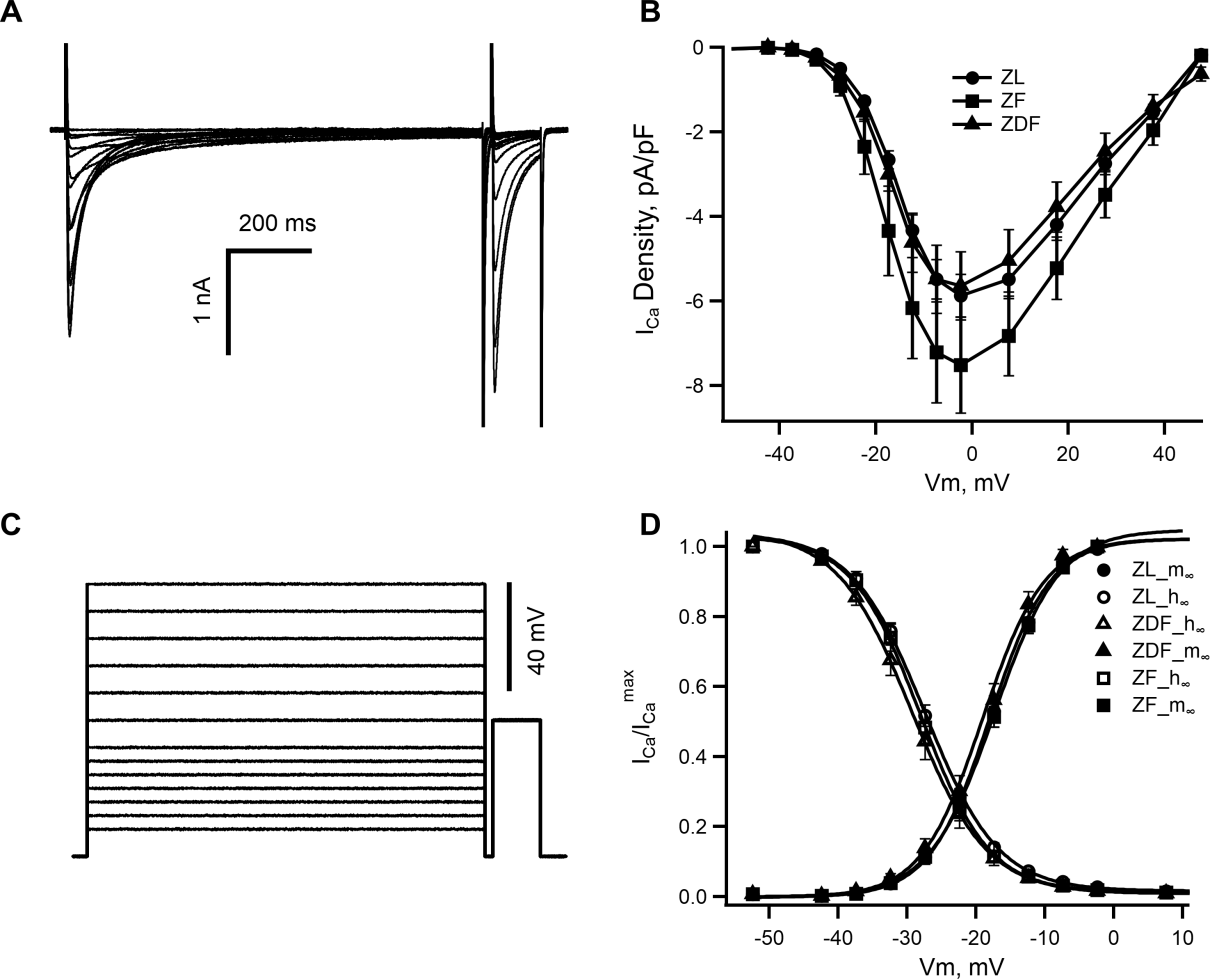
Properties of the L-type Ca^2+^ current (I_(Ca,L)_) in myocytes from ZL, ZF and ZDF rats. **(A) **Typical records of I_(Ca,L)_ from a left ventricular myocyte of a ZF rat. (**B)** ‘I_(Ca, L)_ –V_m_’ relationships in ZL, ZF and ZDF rats. Data points represent mean ± SEM; *n* = 15 ZL, 15 ZF and 13 ZDF cells from 4 ZL, 6 ZF and 5 ZDF rats. (**C)** An illustration of the voltage clamp protocol used for evaluation of the I_(Ca L)_ characteristics. (**D) **A superposition of steady-state activation and inactivation curves reveals similar voltage dependence of the I_(Ca,L)_ gating in ZL, ZF and ZDF rats.

L-type Ca^2+^ current (I_(Ca,L)_) was recorded as described in our previous paper [36]. In brief, I_(Ca,L)_ was recorded using the above patch clamp system. The analogue signal was filtered using a four-pole Bessel filter with a bandwidth of 5 kHz and digitised at a sampling rate of 10 kHz. Borosilicate glass patch pipettes were fabricated from filamented BF150-86-10. Electrode resistances in these experiments ranged from 3 to 5 MΩ and seal resistance from 1 to 5 GΩ. The whole cell bath solution contained the following (in mmol/l): NaCl (145), MgCl_2_ (2), CaCl_2_ (2), HEPES (10) and glucose (10; pH 7.35). The pipette solution contained the following (in mmol/l): CsCl (140), MgCl_2_ (2), TEA Cl (10), EGTA (10), HEPES ( 10) and MgATP (1; pH 7.25). More than 75% of the series resistance was compensated using the Axopatch 200B cell capacitance and series resistance compensation circuitry. Experiments were performed at room temperature. The current–voltage relationship and steady-state inactivation were obtained by applying 1000 ms test pulses in the range of −40 mV to +50 mV from a holding potential of −50 mV. After a brief repolarization to −50 mV, a 120 ms pulse to 0 mV was applied to measure steady-state inactivation, as shown in Figure 1C. The amplitudes of the currents were normalized to the cell membrane capacitance to obtain current densities (pA/pF).

### Data analysis and statistics

**Figure 2:**
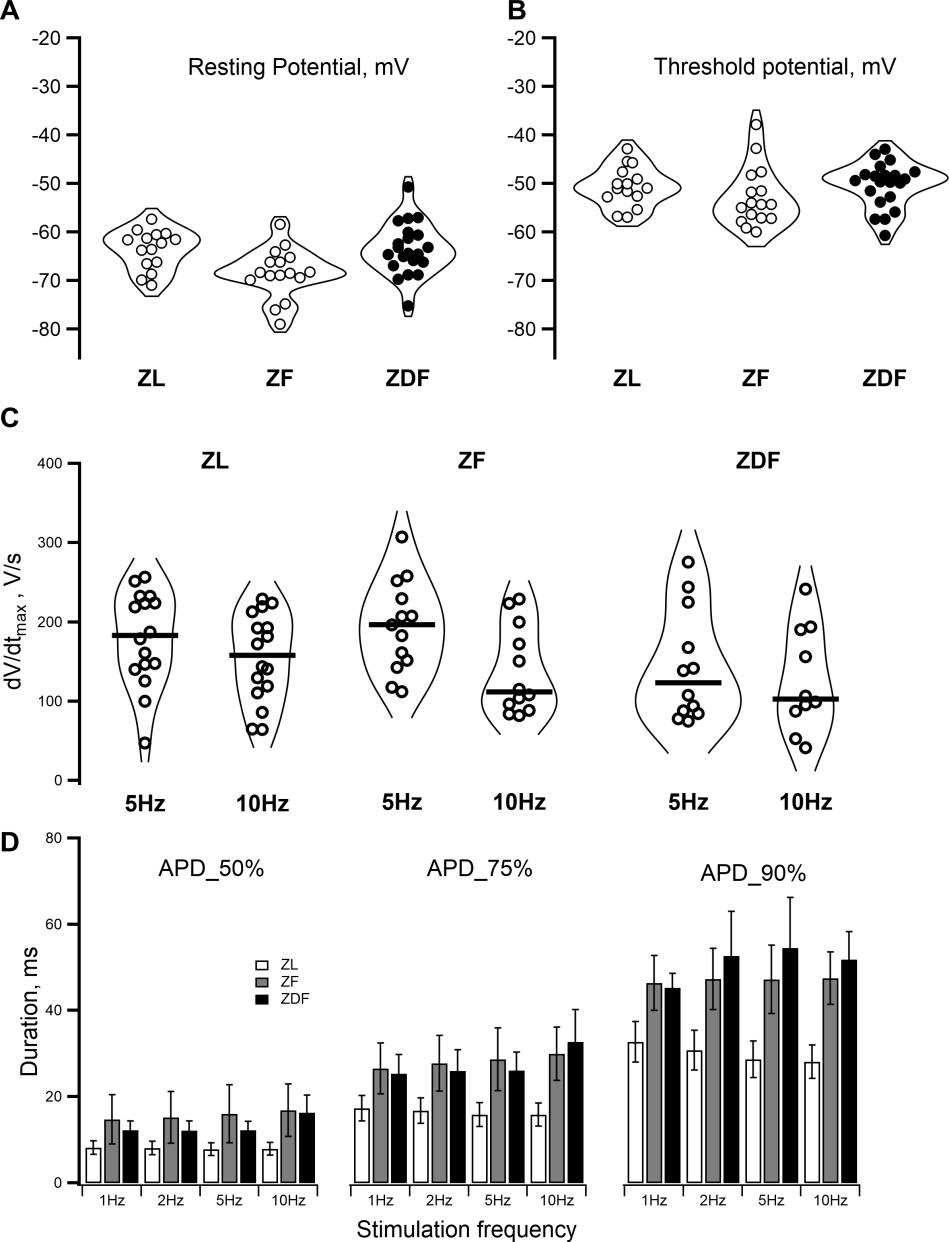
Parameters of resting and action potentials in myocytes from ZL, ZF and ZDF rats. **(A)** The violin plot of the pre-existent resting potential values in myocytes from ZL (left), ZF (middle) and ZDF (right) rats. The widest part of each violin corresponds to the highest distribution density of individual data points. ZF rats had more negative resting potential than the other two groups. (**B)** The violin plots of threshold potentials in ZL, ZF and ZDF rats. Threshold potential was measured as minimal depolarization evoked by a 1 s stimulating current pulse at which an action potential was triggered. Stimuli of lower intensity eliciting only passive electronic responses were considered subthreshold. For all subsequent recordings made in any particular cell, the 1.5 times stronger suprathreshold stimuli were used. (**C)** The violin plots of the action potential upstroke velocity (dV/dt _max_) at 5 H and 10 Hz pacing rate in ZL, ZF and ZDF rats. Bold horizontal lines in each violin denote the median of each data set. The largest drop in dV/dt_max_ values in response to the increase in stimulation rate from 5 Hz and 10 Hz was observed in ZF rats. (**D)** Ventricular myocytes from ZF and ZDF rats show increased duration of the evoked action potentials. The myocytes were paced at 1, 2, 5 and 10 Hz, and action potential duration was measured at 50%, 75% and 90% repolarization. White, grey and black bars represent the mean values correspondingly in ZL, ZF and ZDF rats. The error bars represent the standard deviation.

All numerical data were extracted from the recorded traces using Clampfit 10.6 and transferred to IgorPro 9.0 (Wavemetrics, U.S.A.) for further analysis. Transmembrane voltages were corrected for 3.6 mV junction potential in I_(Ca,L)_ records and 15 mV in I_Na_ records. IgorPro was used to create figures and to perform all statistical calculations. Additional details on the extraction and analysis of specific parameters are provided in the figure legends, where applicable. The Kruskal–Wallis test was used to analyse data with a non-Gaussian distribution (as presented in Figure 2A–C). For normally distributed data, a one-way ANOVA followed by Tukey *post hoc* analysis was used for mean comparisons between the three groups of animals, except for data presented in Figure 2D where a one-way repeated measurement ANOVA was employed. The null hypothesis was rejected, and the difference was considered statistically significant at *P* < 0.05. The ‘*n*’ numbers refer to cell numbers recorded from at least four animals per group.

## Results

### General characteristics of animal groups

The animals were obtained from Charles River at 5 weeks of age and kept at the College of Medicine Animal House for 20–25 weeks before the experiments, allowing for a sufficiently long exposure of the heart to obesity and/or diabetes. After this time, the ZF and ZDF rats exhibited significantly (*P* < 0.05) increased body weight and heart weight compared with ZL rats. Non-fasting blood glucose was significantly elevated in ZDF (384.44 ± 20.28 mg/dl) compared with ZF (140.00 ± 4.58 mg/dl) and ZL (118.25 ± 2.62 mg/dl) rats. Non-fasting blood glucose was not significantly (*P* >0.05) different between ZF and ZL rats. Heart weight/body weight ratio was significantly (*P* < 0.05) increased in ZF (3.27 ± 0.09 mg/g) compared with ZL (2.71 ± 0.07 mg/g) and ZDF (2.79 ± 0.05 mg/g) rats. In agreement with a previously published work [39], the blood plasma insulin level was significantly (*P* < 0.05) higher in ZF rats (12.70 ± 1.15 µg/L) compared with ZL (2.09 ± 0.57 µg/L) and ZDF (2.14 ± 0.29 µg/L) rats.

### Comparison of action potential parameters in myocytes from ZF, ZDF and ZL rats

**Figure 3:**
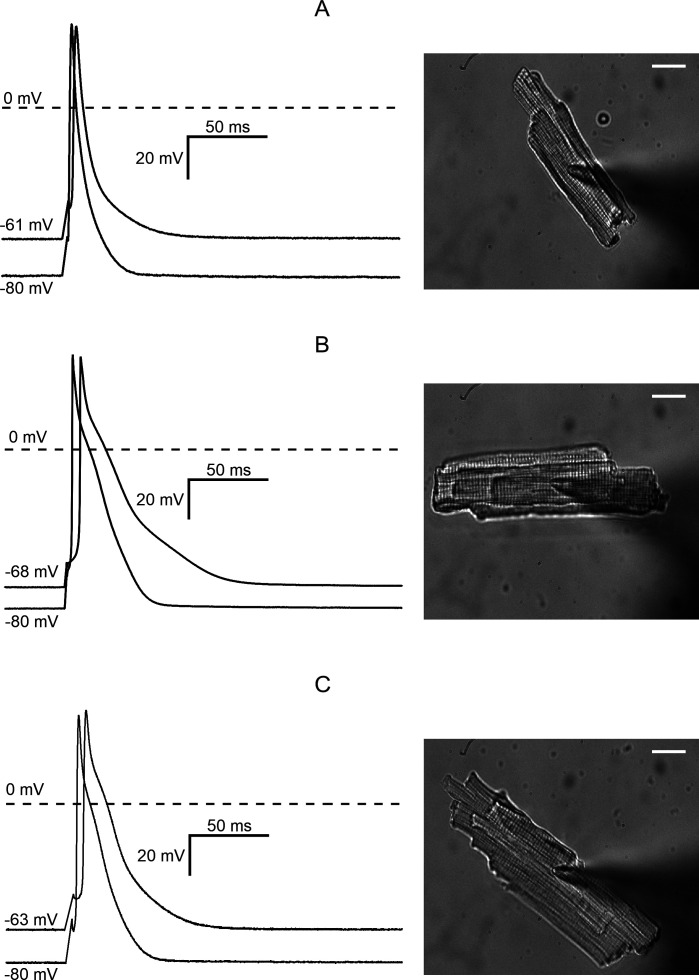
Representative current-clamp recordings from isolated ventricular myocytes. The myocytes shown on the right-hand side of each panel were isolated from (**A**) Zucker Lean (ZL) rats; (**B**) ZF rats, and (**C**) ZDF rats. The left-hand side of each panel shows superimposed action potential tracings evoked by 1 ms threshold current pulse at zero holding current and after applying negative holding current to bring the resting potential to –80 mV. The numbers at the beginning of each trace show the resting potential values before and after the application of holding current. The white bars in each image on the right-hand side represent 20 µm.

In our hands, enzymatic isolation of cardiac myocytes from Zucker rats produced cells with somewhat depolarized RP compared with the normal value measured in adult rat ventricular myocytes. Cells from all groups were capable of generating action potentials with an overshoot in response to brief current pulses of sufficient strength. Typical action potential tracings and a rod-shaped appearance with a clear sarcomere patterning of left ventricular myocytes isolated from ZL, ZF and ZDF groups are shown in Figure 3. The left-hand side panels in Figure 3 show superimposed action potentials elicited from either the pre-existent resting potential or the membrane potential adjusted to −80 mV by applying a constant current. In all three groups of animals, shifting the resting potential to −80 mV resulted in an increased upstroke velocity and shortened the duration of action potentials evoked by suprathreshold stimulation. Averaged values of the RP, holding current required to shift the RP to −80 mV, threshold potential and action potential duration at 50% and 90% repolarization are presented in Table 1. The values of resting and threshold potentials in individual cells are shown in Figure 2A,B as ‘violin’ plots, a convenient tool for visualising the distribution of data in myocytes from ZL, ZF and ZDF groups [40]. The resting potential in myocytes from ZF rats was significantly more negative compared with myocytes from ZL control (*P* = 0.01525), while the difference between the ZDF and ZL groups was not significant. As one would expect, more depolarized cells should have a lower rate of rise of the action potential due to a larger proportion of sodium channels in a state of steady-state inactivation and lower excitability. Indeed, this was found to be the case. As shown in Figure 2C, the upstroke velocity was fastest in myocytes from ZF rats (*P* = 0.04146), while the difference between ZDF and ZL groups was not statistically significant. Concomitantly, the threshold potential was lowest in the ZF group, reflecting their higher excitability (Figure 2B). The upstroke velocity (dV/dt_max_) was dependent on the frequency of stimulation. When paced at 5 Hz, which is close to a physiological heart rate in six-month-old Zucker rats [11], dV/dt_max_ was faster than at a pacing rate of 10 Hz in all groups of animals (Figure 2C). The biggest drop in dV/dt_max_ was observed in ZF rats: from 193 ± 16.1 V/s at 5 Hz to 136 ± 16.0 V/s at 10 Hz (*P* < 0.05, *n* = 13). Notably, ZDF rats showed significantly lower dV/dt_max_ at all stimulation frequencies.

### Sodium current in left ventricular myocytes from ZL, ZF, and ZDF rats

**Figure 4:**
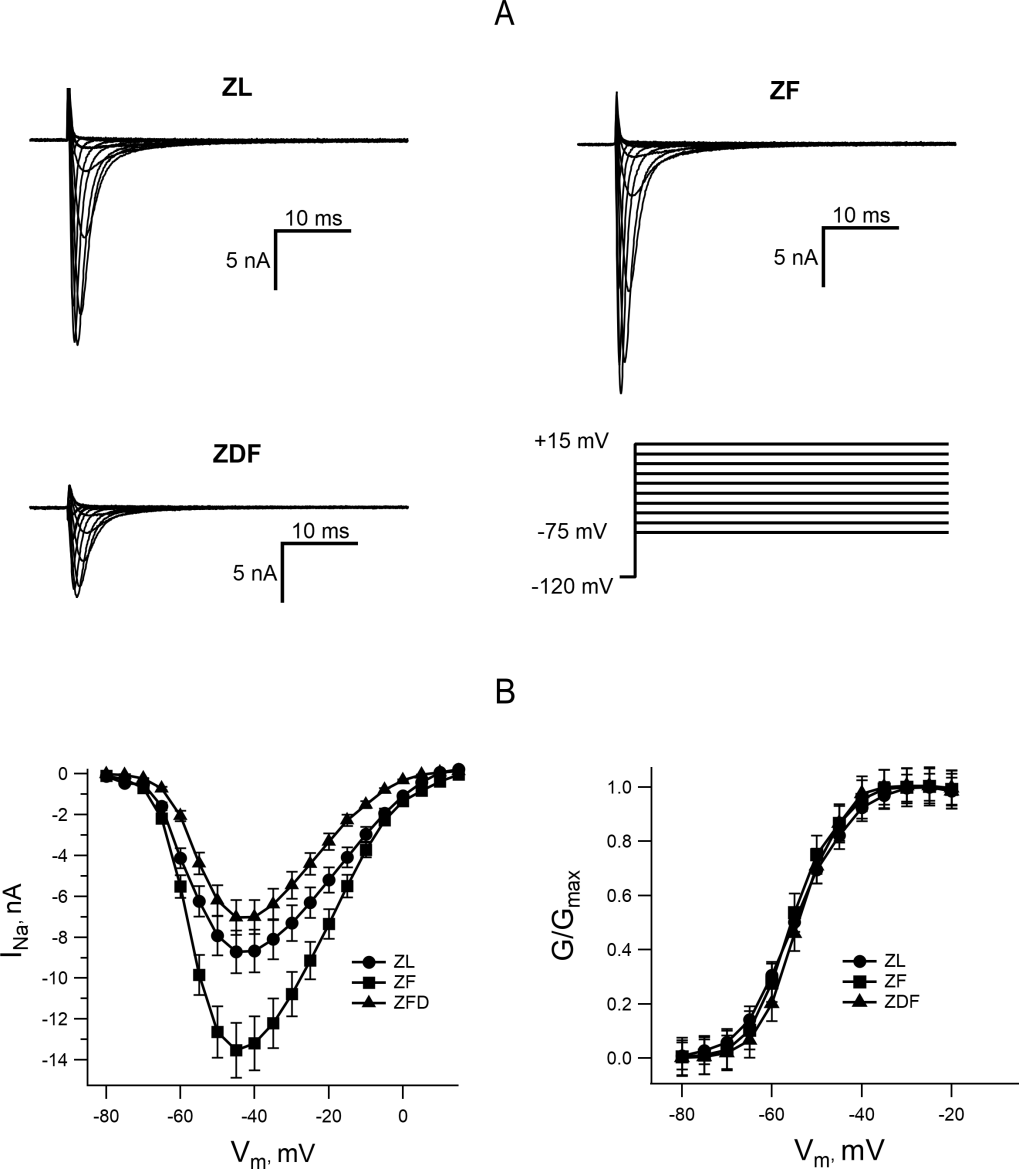
Comparison of voltage-gated Na^+^ currents in myocytes from ZL, ZF, and ZDF rats. **(A)** Representative traces of I_Na_ elicited by depolarizing voltage clamp pulses (the voltage protocol is shown in the lower panel on the right). (**B)** Averaged ‘I_Na_–V_m_’ curves (left-hand side panel) show a significantly increased I_Na_ amplitude in ZF rats in comparison with ZL and ZDF rats. The right-side panel shows the voltage dependence of I_Na_ activation. Sodium conductance (**G**) was derived from the ‘I_Na_–V_m_’ curves on the left and normalized to the maximum (G_max_) in each group of rats. The peak of the ‘I_Na_–V_m_’ curve was significantly higher in the ZF group. There was no shift in the ‘G_Na_–V_m_’ curves along the voltage axis in any of the three groups of animals.

The low dV/dt_max_ in ZDF rats implies that there could be a diabetes-induced decrease in the expression of sodium channels and/or diabetes-induced changes in their gating. We tested this by comparing the amplitudes and voltage dependence of the inward sodium current in myocytes from ZL, ZF and ZDF rats. Figure 4A shows representative traces of I_Na_ recorded in myocytes from the lean, fat and fat diabetic animals. The current waveforms showed similar time and voltage dependence (Figure 4A). In all three groups, the current was activated at potentials above −70 mV and the peak of the ‘I_Na_–V_m_’ curve occurred at −40 mV. There were no statistically significant shifts of the ‘G_Na_–V_m_’ curve along the voltage axis between the three groups of animals (Figure 4B, right-hand side panel). The time constant of inactivation and its voltage dependence were similar in all three groups. The only difference detected in these experiments was that the I_Na_ amplitude was significantly higher in ZF rats compared with ZL control (Figure 4B, left-hand side panel). Such augmentation of the I_Na_ was not observed in the ZDF group. The diabetic rats exhibited a diminished I_Na_ compared with the ZF and ZL groups, which would explain the low upstroke velocity observed in diabetic rats.

Having identified the cause of the diminished upstroke velocity of the ventricular action potential, we next investigated the relationship between action potential duration and stimulation frequency. In ZL rats, the action potential durations measured at 50%, 75% and 90% repolarization were similar at stimulation frequencies ranging from 1 to 10 Hz. However, the fatty and diabetic rats showed statistically significant prolongation of their ventricular action potentials (illustrated in Figure 2D). Interestingly, the duration of repolarization was increased at all voltage levels where it was measured, i.e., at 50%, 75% and 90% of the action potential amplitude, suggesting that the observed elongation was due to the impediment of several ionic currents flowing through the membrane during an action potential.

### L-type Ca^2+^ current in left ventricular myocytes from ZL, ZF and ZDF rats

Multiple studies have described the involvement of several components of voltage-gated K^+^ current as well as voltage-independent inward rectifier K^+^ current in diabetes-induced elongation of ventricular action potential duration. In addition, the contribution from I_(Ca,L)_ ‘window current’ has been suggested, although reports on the I_(Ca,L)_ remodelling in diabetic myocardium remain inconsistent. Hence, we have compared the I_(Ca,L)_ densities and voltage dependence of steady-state activation/inactivation parameters in ventricular myocytes from ZL, ZF and ZDF rats. The double-pulse voltage protocol used in this series of experiments allowed the estimation of steady-state activation and inactivation parameters (Figure 1D) simultaneously with the peak amplitude of I_(Ca,L)_ (Figure 1B). Figure 1A illustrates typical current traces elicited by the voltage pulses shown in Figure 1C. During the initial (conditioning) 1 s pulse, the membrane voltage was stepped between −40 and 0 mV in 5 mV increments and between 0 and +50 mV in 10 mV steps. This evoked inward currents that reached a peak and then inactivated to a steady-state level (see Figure 1A), yielding a current–voltage relationship typical of the voltage-gated L-type Ca^2+^ current. After each 1 s conditioning pulse, the membrane voltage was stepped back to the holding potential for 20 ms and then a 120 ms pulse to 0 mV to measure the fraction of channels remaining available for activation. The successive conditioning pulses were repeated with 20 s intervals between them, which provided sufficient time for full recovery of Ca^2+^ channels from inactivation during the preceding conditioning pulse. The 5 mV increments in conditioning pulses between –40 and 0 mV allowed for a more precise determination of the I_(Ca, L)_ activation threshold and the position of the overlap between steady-state activation and inactivation curves (‘window’ current) along the voltage axis. As shown in Figure 1D, there was no significant difference in the ‘window’ current between myocytes from ZL, ZF and ZDF rats. The voltage dependence of the I_(Ca,L)_ was nearly identical between the three groups of animals as judged by Boltzmann’s fit to the steady-state activation and inactivation curves (not illustrated). Likewise, the maximum densities of the L-type Ca^2+^ currents were identical in myocytes from ZL and ZDF rats. There was a trend towards an I_(Ca, L)_ increase in the ZF rats although this did not reach statistical significance (*P* = 0.06375).

## Discussion

The Zucker rat model has proven to be one of the most useful small animal models for studying the impact of T2DM on the cardiovascular system [7,8,10–12]. While many studies have used the Zucker rat model to explore the role of metabolic factors and biochemical processes in the pathogenesis of T2DM [4,19], there is a paucity of electrophysiological data available [4,12,17]. Yet, the electrophysiological approach is indispensable for the mechanistic understanding of the obesity and T2DM-related abnormalities of cardiac impulse propagation and the development of cardiac arrhythmias. In this study, we investigated the remodelling of left ventricular action potentials in ZL, ZF, and ZDF rats. Our results show the prolongation of ventricular action potentials in ZF and ZDF rats at 1–10 Hz pacing frequencies. These results are consistent with findings in the literature [25]. The pathophysiological significance of this phenomenon is that the prolonged action potential duration may facilitate the development of early afterdepolarizations, which can induce triggered activity in the ventricle, as observed in ECG recordings such as *torsades des pointes*, a specific type of polymorphic ventricular tachycardia that can lead to sudden cardiac death. Multiple electrophysiological mechanisms have been implicated in long QT syndrome. A well-established view is that cardiac delayed rectifier voltage-gated and voltage-independent inward rectifier potassium channels play a pivotal role in this phenomenon. More recently, the contribution of the L-type Ca²^+^ ‘window current’ and the resurgence of the late Na^+^ current to the development of prolonged action potentials and early afterdepolarizations has been explored. We investigated whether the biophysical properties of the L-type Ca^2+^ channels were altered in ZF and ZDF rats compared with control ZL rats and found that this was not the case. In the Zucker rat, neither L-type Ca^2+^ channel gating nor I_(Ca, L)_ density is affected by obesity or diabesity. Our experiments produced no evidence for a non-inactivating sodium current. Furthermore, the I_Na_ inactivation kinetics were similar in all three animal groups, ruling out the possibility that the late component of I_Na_ played a role under our experimental conditions. However, we cannot fully reject the possibility of late sodium current appearance under specific hormonal conditions, e.g., in the presence of high levels of angiotensin and/or aldosterone, as found in hypertensive diabetic individuals. The novelty of our study lies in the use of the Zucker rat model, which enabled us to uncover the distinct effects of obesity and T2DM on excitability and the action potential upstroke velocity: a lower threshold and some acceleration of the dV/dt_max_ in obese rats vs. a higher threshold and a noticeable reduction in the dV/dt_max_ in diabetic animals (Figure 2B and C). Furthermore, the results of our patch-clamp experiments revealed that the observed difference in dV/dt_max_ between ZF and ZDF rats was due to increased I_Na_ amplitude in the fatty non-diabetic group and diminished I_Na_ expression in their diabetic counterparts.

The limitation of our study is that we have to use a reduced extracellular sodium concentration to maintain adequate voltage control, and our experiments were conducted on isolated cells, while in real life, the action potential propagation involves multicellular tissue of complex architecture. Nevertheless, our findings are compatible with the idea that a previously described impairment of electrical conduction in diabetic myocardium is, in large part, due to the diminished expression of I_Na_ in ventricular myocytes of diabetic animals [41]. Indeed, targeted transgenic disruption of cardiac sodium currents in mice resulted in impaired AV node and ventricular conduction. Similar results were obtained in a rabbit model of T1DM. Taken together, these findings suggest that dysfunction of myocardial sodium channels is a major cause of impaired electrical conduction.

The differential effects of obesity *per se* and obesity with diabetes on the action potential upstroke velocity suggest that diabetic individuals, in contrast with their obese non-diabetic counterparts, could be at a higher risk of developing re-entry arrhythmias and conduction block.

In conclusion, the results of our study show a substantial and dissimilar remodelling of the ventricular action potential waveforms in obese non-diabetic and obese diabetic animals underlined by the differential up- and down-regulation of I_Na_ in ventricular myocytes.

**Clinical perspectives**

- Obesity predisposes to the development of metabolic syndrome, insulin resistance and T2DM. The majority of T2DM patients are obese, although not all obese individuals develop T2DM. It remains unclear whether obesity per se and T2DM induce distinct impacts on the myocardium.
- Using the Zucker rat model of obesity and T2DM, we identified the differential effects of obesity and T2DM on myocardial excitability and the ventricular action potential upstroke velocity: a lower threshold and some acceleration of the dV/dt_max_ in obese rats vs. a higher threshold and a noticeable reduction in the dV/dt_max_ in diabetic animals.
- Our data suggest that the treatment of cardiac dysrhythmias in obese diabetic and obese non-diabetic hearts should be different.

## Data Availability

Data will be provided upon reasonable request

## Open Access

This article has been published open access under our Subscribe to Open programme, made possible through the support of our subscribing institutions, learn more here: https://portlandpress.com/pages/open_access_options_and_prices#conditional

## Abbreviations

Cx43: connexin 43
DM: diabetes mellitus
T2DM: type 2 diabetes mellitus
ZDF: Zucker diabetic fatty
ZF: Zucker fatty
ZL: Zucker lean
fa: fatty
